# Prevalence of abdominal obesity and its correlates among adults in a peri-urban population of West Africa

**DOI:** 10.3934/publichealth.2019.3.334

**Published:** 2019-09-23

**Authors:** Simone K. Malik, Jerome Kouame, Mory Gbane, Madikiny Coulibaly, Michèle D. Ake, Odile Ake

**Affiliations:** 1 Unit of Cardiology, National Institute of Public Health, Abidjan, Ivory Coast; 2 Department of Public Health, Hydrology and Toxicology, Faculty of Pharmaceutical and Biological Sciences, University Felix Houphouet Boigny, Abidjan, Ivory Coast; 3 Nutrition Department, National Institute of Public Health, Abidjan, Ivory Coast; 4 Maternal and Child Health, National Institute of Public Health, Abidjan, Ivory Coast; 5 Pharmacy-Nutrition Laboratory, National Institute of Public Health, Abidjan, Ivory Coast

**Keywords:** abdominal obesity, noncommunicable diseases, peri-urban areas, Ivory Coast, Africa

## Abstract

**Introduction:**

Prevalence of abdominal obesity dramatically increases both in developed countries and in developing countries. In several regions in Africa, obesity (especially abdominal) is seen as a sign of affluence, dignity and respect. The objective of this study was to determine prevalence of abdominal obesity and seek some factors associated in a peri-urban population of West Africa.

**Methods:**

During April-May 2014, a cross-sectional study was conducted in Anonkoi 3, a neighborhood of Abidjan (Ivory Coast). Adults of 18 years old and over, not bedridden were included. Sampling was done in two stages. First, we selected the households. Then in each household we randomly chose one adult. Abdominal obesity was measured using a measuring tape and defined by waist circumference ratio (TT) to hip circumference (TH) greater or equal to 0.80 in women and greater or equal to 0.95 in men. Data from sociodemographic, corpulence, socioeconomic level, food habit, level of physical activity and blood pressure were analyzed. Univariate analysis using the Pearson KHI-square test at a significance level of 0.05 and a logistic regression was performed.

**Results:**

We visited 486 households in which 486 people aged 36.1 ± 12.83 years agreed to participate in the study. Prevalence of abdominal obesity was 50.8%. Those aged 30–45 years, women, couples, those with a primary level of education, the poor, people with high blood pressure, subjects less active and obese (general obesity) had more abdominal obesity. After logistic regression, individuals aged 30–45 years (adjusted OR = 2.35; p = 0.004) and 45 years and older (adjusted OR = 3.18; p = 0.001); females (adjusted OR = 49.05; p = 0.000); hypertension (adjusted OR = 2.26; p = 0.014) and obesity (OR = 2.94; p = 0.009).

**Conclusion:**

This work allowed us to estimate a relatively high prevalence of abdominal obesity in a peri-urban African population.

## 1. Introduction

The increasing prevalence of obesity in past decades among several countries has been identified as a global pandemic [1]. Every year, 1.8 million people die prematurely from non-communicable diseases related to overweight or obesity [2] and it is estimated that 38% of the world adult population will be overweight and 20% obese by 2030 [3].

The general obesity was established as a cardiometabolic risk factor, however, abdominal obesity is recognized as the best predictor of this risk regardless of body mass index [4],[5]. The prevalence of this form of obesity is increasing dramatically in developed countries as well as in developing countries [6]–[8] due to urbanization and the rising westernization of the population's lifestyle [9].

In several regions in Africa, obesity (especially abdominal) is seen as a sign of affluence, dignity and respect [10],[11]. It is more and more common to find abdominal obesity in young adults. The data of obesity prevalence have been estimated more for general obesity than abdominal obesity. Some authors have reported the prevalence of abdominal obesity in the literature. In Uganda, Kabwama [12] reported a prevalence of 11.5% in a sample of 4900 adults while in Nigeria, Amole found a prevalence of 33.8% [8].

In each study, the prevalence of abdominal obesity varied according to the types of population, age of respondents and the environments in which these studies were conducted. The peri-urban environment has the particularity of bringing together populations who have urban and rural habits and lifestyles. This environment is generally dynamic, resulting from the mixing between the surrounding urban and rural areas. The population growth rate is high due to migration to cities [13].

In Ivory Coast, some authors have estimated the prevalence of abdominal obesity. Hauhouot-Attoungbré et al. reported a prevalence of 30% among lactating women in post-partum period [14]. Kouakou et al. noted a prevalence of 19.56% among students [15]. None of these studies were specific to the peri-urban environment. The objective of this study was to determine the prevalence of abdominal obesity and seek some factors that are associated in a peri-urban population of West Africa.

## 2. Methods

### 2.1. Framework of the study

This study was conducted in Abidjan (Ivory Coast) in the neighborhood of Anonkoi 3, a village in the commune of Abobo. Abobo is the second most populated municipality in the district of Abidjan, with a density of 167 inhabitants per square kilometer [16]. Abobo has 28 districts and villages including more than ten shantytowns and slums. These neighborhoods are, for the most part not serviced. Anonkoi 3 is unserviced village of Abobo [17].

### 2.2. Sampling

This was a cross-sectional study from April 24^th^ to May 23^rd^ 2014. For the calculation of the sample size, in the absence of a previous estimate sufficiently representative of the prevalence of abdominal obesity in the Ivorian population, we used the prevalence of overweight and obesity (p = 32.2%). The risk of error and accuracy was 5%. Thus, the sample size calculated was 336. Assuming a response rate of 80%, the minimum sample size was estimated at 420.

### 2.3. Sampling strategy

The neighborhood of Anonkoi 3 is a village in the commune of Abidjan. In this neighborhood households are not numbered. In the general census of the population in 1998, the neighborhood had 474 households [18]. However during a comprehensive study in this area, Sackou Kouakou et al. identified 668 households [16]. Therefore, we conducted a random sample, we calculated a sampling interval of two (668/336 = 1.98). We considered household No. 1 the first household found when we had access to the area, and we visited one in two households. Sampling was done in two stages. First, we selected the households. Then in each household we randomly chose one adult.

### 2.4. Study population

Included in this study were all adults of 18 years old and over, not bedridden and present at the time of the survey. Not included were women in pregnancy or breastfeeding. In each household visited, an adult of 18 years old and over was selected. In the presence of more than one adult of 18 years old and over, only one person was selected randomly.

### 2.5. Data collection

Data collection was performed from a pre-tested questionnaire following free and informed consent of the selected person (written or oral consent). The data collected were of various kinds:

1. Sociodemographic data (age, sex, marital status, level of study).

2. Abdominal obesity was measured using a measuring tape and is defined by a waist circumference ratio (TT) to hip circumference (TH) greater than 0.80 in women and greater than 0.95 in men [19].

3. Corpulence was defined from the Body Mass Index (BMI) [20]. The size was measured using a Fiber-Glass® measuring rod and the weight using a Camry® brand scale model scal160 that can support up to 160 kg. Scale (Considered obese were, individuals with a BMI greater than or equal to 30 kg/m^2^ and not obese, those with a BMI less than 30kg/m^2^).

4. Socioeconomic level was evaluated through the score of poverty or wealth index. The index was calculated using data on the possession of material goods by households (e.g. televisions, bicycles, cars, materials used for the construction of housing, types of access to water and sanitation). The scale of relative wealth was then classified into five categories (the poorest, the poorer, the middle, the richer and the richest) in the sample quintile [21].

5. The food habit was described by snacking. Snacking has been defined as the act of taking at least an extra meal outside of regular meals (breakfast, lunch or dinner).

6. The level of physical activity was assessed in three categories (low active, active, and very active) by the International Physical Activity Questionnaire (IPAQ) in its shortened version. Then the categories “active and very active” were grouped into a single category called “active”. Thus, the level of physical activity was divided into “less active” and “active”. The IPAQ questionnaires explores the intensity of physical activity (vigorous, moderate, low), frequency (days per week) and duration (hours/minutes per day). The IPAQ 2002 considers vigorous activities, those that require physical effort and that make the strongest breathing (heavy lifting, aerobics, pedaling quickly). Moderate activities are those that require intermediate physical exertion (lifting light objects, pedaling regularly, playing tennis).

7. Blood pressure (BP) was measured by an OMRON^R^ M6 brand electronic blood pressure monitor with an armband. Three measurements were taken after five minutes of rest. Considered to have high blood pressure people whose systolic blood pressure was higher than 140 mmHg or diastolic blood pressure greater than 90 mmHg.

### 2.6. Data analysis

Data was entered in Epi data software (version 3.1) and analyzed using the R software version 1.1.447 studio. The search of factors associated with obesity was done in two stages. First, we performed a univariate analysis using the Pearson KHI-square test at the 0.05 significance level. Then, the variables having a value less than 0.05 p were included in a logistic regression model. The adjusted odds ratio and the confidence intervals were calculated at 95%.

### 2.7. Ethical considerations

The survey participants were informed about the reasons for the study. Those who could read and write were agreed to fill out a personal identification form. For those who had no educational level (who could neither read nor write) oral consent was obtained. They then agreed to submit to the taking of the parameters. Their free and informed consent was obtained prior to the survey. They were free to withdraw at any time without prejudice to the investigation. Data was collected in an anonymous way.

### 2.8. Results

We visited 486 households in which 486 adults of 18 years old and older (one person per household) agreed to participate in the study. There were 327 women and 159 men, with a sex ratio (M/F) of 0.48. The average age of our population was 36.1 ± 12.83 years old. Almost half of the population lived in pairs (couple). We had about 2 in 5 with a secondary level of study and 1 in 3 had no educational level (did not go to school). In this environment, the poorest accounted for 1/3 of the population, and there were as many people snacking as those who were not snacking. More than a quarter of the population had high blood pressure and a little over two-fifths (2/5) of those were less active. The prevalence of abdominal obesity was 50.8% or more than half the population.

**Table 1.** Population characteristics and univariate analysis of factors associated with abdominal obesity.

	Numbers n = 486 (%)	abdominal obesity n = 247 (%)	No abdominal obesity N = 239 (%)	gross OR	95%	P value
Age (years)						0.000
15–30	184 (37.8)	74 (30.0)	110 (46.0)	1		
30–45	171 (35.2)	106 (42.9)	65 (27.2)	2.42	[1.58 to 3.72]	
45–and more	131 (27.0)	67 (27.1)	64 (26.8)	1.56	[0.62 to 2.62]	
						
Sex						0.000
Women	327 (67.3)	236 (95.5)	91 (38.1)	34.89	[18.83 to 71.11]	
Man	159 (32.7)	11 (4.5)	148 (61.9)	1		
						
Marital status						0.000
Only	221 (45.5)	92 (37.2)	129 (54.0)	1		
In a relationship with	265 (54.5)	155 (62.8)	110 (46.0)	1.97	[1.37 to 2.83]	
						
Study level						0.000
No	155 (31.9)	103 (41.7)	52 (21.8)	6.85	[3.48 to 14.30]	
Primary	83 (17.1)	56 (22.7)	27 (11.3)	7.18	[3.40 to 15.97]	
Secondary	190 (39.1)	75 (30.4)	115 (48.1)	2.26	[1.17 to 4.62]	
Superior	58 (11.9)	13 (5.2)	45 (18.8)	1		
						
Score of poverty						0.000
poorest	162 (33.3)	96 (38.9)	66 (27.6)	2.02	[1.14 to 3.64]	
Poorer	83 (17.1)	52 (21.1)	31 (13.0)	2.33	[1.21 to 4.55]	
Middle	72 (14.8)	27 (10.9)	45 (18.8)	0.84	[0.42 to 1.65]	
Richer	102 (21.0)	44 (17.8)	58 (24.3)	1.06	[0.57 to 1.98]	
Richest	67 (13.8)	28 (11.3)	39 (16.3)	1		
						
Snacking						0.785
Yes	244 (50.2)	126 (51.0)	118 (49.4)	1.06	[0.74 to 1.52]	
No	242 (49.8)	121 (49.0)	121 (50.6)	1		
						
Arterial pressure						0,007
HTA	140 (28.8)	85 (34.4)	55 (23.0)	1.75	[1.17 to 2.62]	
No HTA	346 (71.2)	162 (65.6)	184 (77.0)	1		
						
Physical Activity Level						0.000
Assets	285 (58.6)	117 (47.4)	168 (70.3)	1		
Few assets	201 (41.4)	130 (52.6)	71 (29.7)	2.62	[1.81 to 3.82]	
						
Obesity						0.000
Yes	72 (14.8)	59 (23.9)	13 (5.4)	5.46	[2.99 to 10.97]	
No	414 (85.2)	188 (76.1)	226 (94.6)	1		

Table 1 shows the distribution of the population by various factors as well as associations between abdominal obesity and these factors. We found a link between abdominal obesity and all the factors sought outside snacking. Indeed, abdominal obesity is mostly observed in aged 30 to 45 subjects. They were significantly two to three times more likely to have abdominal obesity compared to those aged between 15 and 30 years old (OR = 2.42; 95% CI, p = 0.000). Women were almost thirty-five [35] times more likely to have abdominal obesity compared to men (OR = 34.89, 95% CI; p = 0.000). People in couples were about twice as likely to have abdominal obesity compared to those individuals who were not (OR = 1.97; 95% CI; p = 0.000). Those with a primary level of study were seven to eight times more likely to have abdominal obesity compared to those with a higher level of study (OR = 7.18; 95% CI, P = 0.000). The poorer (the less affluent people) were almost two and half times more likely to have abdominal obesity compared to the richest (OR = 2.33; 95% CI; p = 0.000). The people with high blood pressure were almost twice as likely to have obesity compared to people without hypertension (OR = 1.75; 95% CI, p = 0.007). The less active participants were two to three times more likely to have abdominal obesity relative to assets (OR = 2.62; 95% CI, p = 0, 000), and those obese (general obesity) had five to six times more likely to have abdominal obesity compared with non-obese (OR = 5.4; 95% CI, P = 0.000). There was no relationship between snacking and abdominal obesity.

**Table 2.** Multivariate analysis of factors associated with abdominal obesity.

Variable	Adjusted OR (95% CI)	P value
Age		
30–45	2.35 (1.30 to 4.29)	0.004**
45–and more	3.18 (1.56 to 6.78)	0.001**
		
Sex		
Women	49.05 (22.82 to 116.83)	0.000***
		
Marital status		
In a relationship with	1.53 (0.90 to 2.60)	0.115
		
Study level		
No	1.30 (0.47 to 3.60)	0.606
Primary	1.94 (0.68 to 5.61)	0.215
Secondary	0.82 (0.32 to 2.14)	0.689
		
Score of poverty		
The poorest	1.20 (0.53 to 2.73)	0.648
Poor	1.08 (0.43 to 2.72)	0.860
Rich	0.52 (0.21 to 1.3)	0.174
Richest	1.16 (0.48 to 2.78)	0.735
		
Snacking		
Yes	0.73 (0.44 to 1.22)	0,235
		
Arterial pressure		
HTA	2.26 (1.19 to 4.46)	0,014*
		
Physical Activity Level		
Few assets	1.11 (0.66 to 1.85)	0.687
		
Obesity		
Yes	2.94 (1.34 to 7.02)	0.009**

Table 2 shows the results of the multivariate analysis. Factors associated independently with abdominal obesity were: age 30 to 45 years (adjusted OR = 2.35; 95% CI; p = 0.004) and 45 years and over (adjusted OR = 3.18; 95% CI; p = 0.001); females (adjusted OR = 49.05, 95% CI; p = 0.000); hypertension (adjusted OR = 2.26; 95% CI; P = 0.014) and obesity (OR = 2.94; 95% CI; P = 0.009). Marital status, level of study, the score of poverty and the level of physical activity were however no longer significantly associated with abdominal obesity.

## 3. Discussion

This study, conducted in a peri-urban area, aimed to determine the prevalence of abdominal obesity, and seek some associated factors in a population of West Africa. It reveals that 50.8% of the population, or one in two adults had abdominal obesity. This prevalence is higher than those reported in some countries in West Africa [8],[22],[23]. This difference in prevalence may be due to the measurement technique. In fact, these studies have defined abdominal obesity as waist circumference, whereas in our study abdominal, obesity was defined as the turn ratio of waist/hip circumference. Abdominal obesity contributes benefit to cardiometabolic risk, due to intra-abdominal accumulation of visceral fat and is associated with the appearance of multiple cardiometabolic diseases, regardless of body mass index [24]. So, to Anonkoi 3, one in two adults is at high cardiometabolic risk.

Our investigation found that abdominal obesity mainly concerned the age of 30 and 45 years old, and women who were about 35 times more likely to have abdominal obesity than men. Some studies have reported that abdominal obesity was more common between 40 to 49 years old [8] or after 40 years old [25],[26], and that abdominal obesity was more common in women (8.26 to 28) compared to men. This difference in prevalence of abdominal obesity between women and men has been attributed to several factors including: the difference of sex steroid hormones that cause the divergence of the structure and body composition, particularly in adolescence [30]. The difference between the environment and the genetic susceptibility of the accumulation of fat between men and women [31]. This difference in prevalence may also be explained by gender [32]: pregnancy resulting in an increase in visceral fat and postpartum abdominal [33] and postmenopausal redistribution of body fat in the abdominal area [34]. The types of daily activities and cultural problems between the sexes could also be one possible explanation for this result [35]. Therefore, to Anonkoi 3, women between 30 and 45 years old have a high cardio-metabolic risk.

In this peri-urban environment, abdominal obesity was more common among people living in couples. The literature on the association between abdominal obesity and living alone or in couples is contradictory. Some studies have found similar results to ours [35],[36]. While other surveys have instead noted a link between abdominal obesity and living alone [25],[28]. The fact that people in couples are more obese abdomen was attributed to a change in eating habits after marriage and increased social support [12].

At Anonkoi 3, those with a primary level of study were more likely to have abdominal obesity. This is contradictory to those of several studies in the literature [12],[28] which reported rather frequent abdominal obesity in people with higher education levels. In their respective contexts, these authors (Kabwama and Munyogwa) likened having a high level of education to better socio-economic status and therefore being free from need. However, our results corroborate those of Yoo [7]. We could explain these results by the fact that people with low levels of education are not very concerned of the consequences of their condition, especially that they do not receive any formal education or awareness on cardiovascular risk factors.

We also noted that people with high blood pressure were more likely to have abdominal obesity than those without hypertension. The link between abdominal obesity and high blood pressure is well established. This observation was made by several authors [12],[38],[39]. The severity of obesity-related diseases is not directly associated with total body fat accumulation, but its distribution, particularly to visceral localization. There is a distinction between the metabolic function of the central or abdominal obesity (visceral abdominal), and peripheral (subcutaneous) [40],[41]. Visceral adipose tissue is adipose tissue which is stored in the abdominal cavity around the internal organs, it communicates with other central and peripheral organs by the synthesis and secretion of a multitude of molecules generally referred to as adipokines. The accumulation of visceral adipose tissue induces chronic inflammation and metabolic disorders such as high blood pressure [42].

At Anonkoi 3, less active people were more likely to have abdominal obesity. These results are consistent with several studies that state that regular physical activity has a beneficial effect on abdominal obesity [41]–[43]. In a much greater extent than dietary excess, inactivity appears to be an independent risk factor and strong for visceral fat accumulation [45].

In our study, we used the waist-to-hip ratio. However, it is more common to use the abdominal diameter as a measure of abdominal obesity. Moreover, among the metabolic disorders associated with abdominal obesity, only the influence of arterial hypertension has been sought.

**Study limitations:** We noted some limitations to our study. These limitations were about food habits, women parity, sampling strategy and population ethnicity. Food habits and lifestyles play a major role in abdominal obesity. In our study, these habits have only been described as snacking. The parity of women has not been sought although, it is known that female parity influences abdominal obesity.We used sampling interval while houses were not officially numbered. Although several authors have noted that abdominal obesity is a sign of ease and respect, we should have researched in our study, the influence of ethnicity on abdominal obesity

## 4. Conclusion

This work allowed us to estimate a relatively high prevalence of abdominal obesity in a peri-urban African population. This type of obesity was more common in women and is associated with known risk factors for cardiovascular disease. These results show the interest of raising awareness for a healthier lifestyle.
